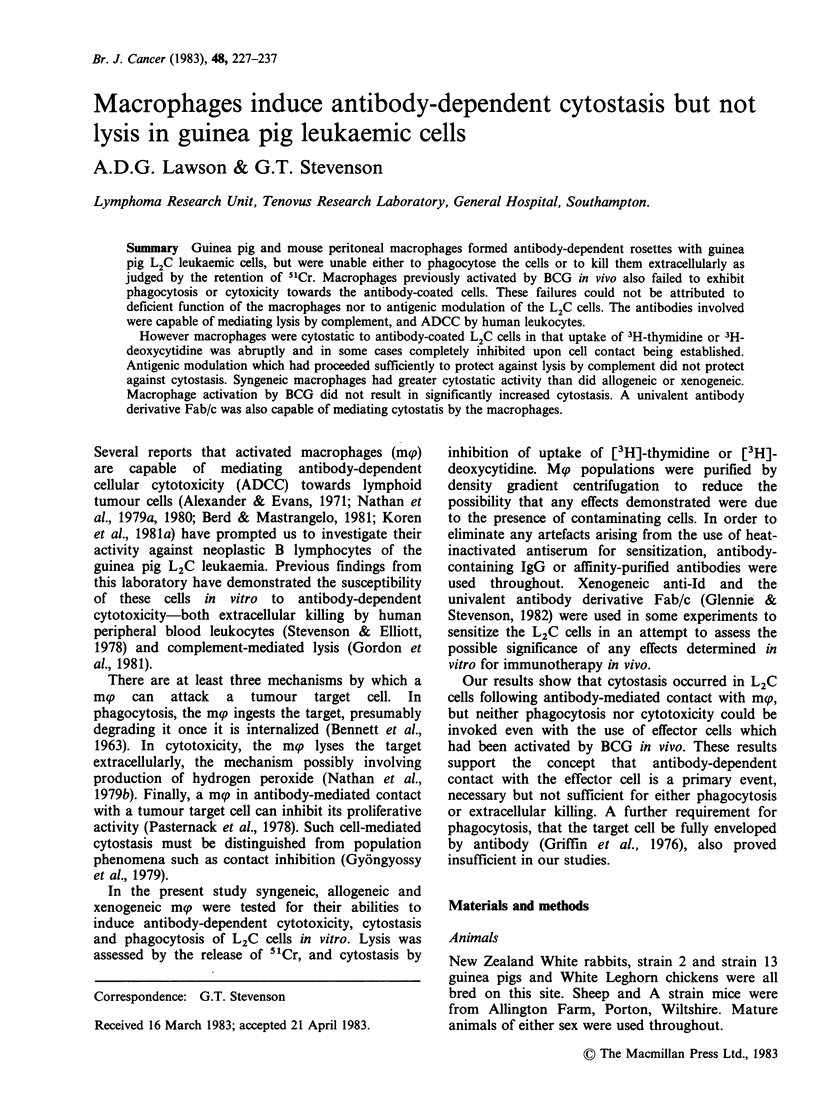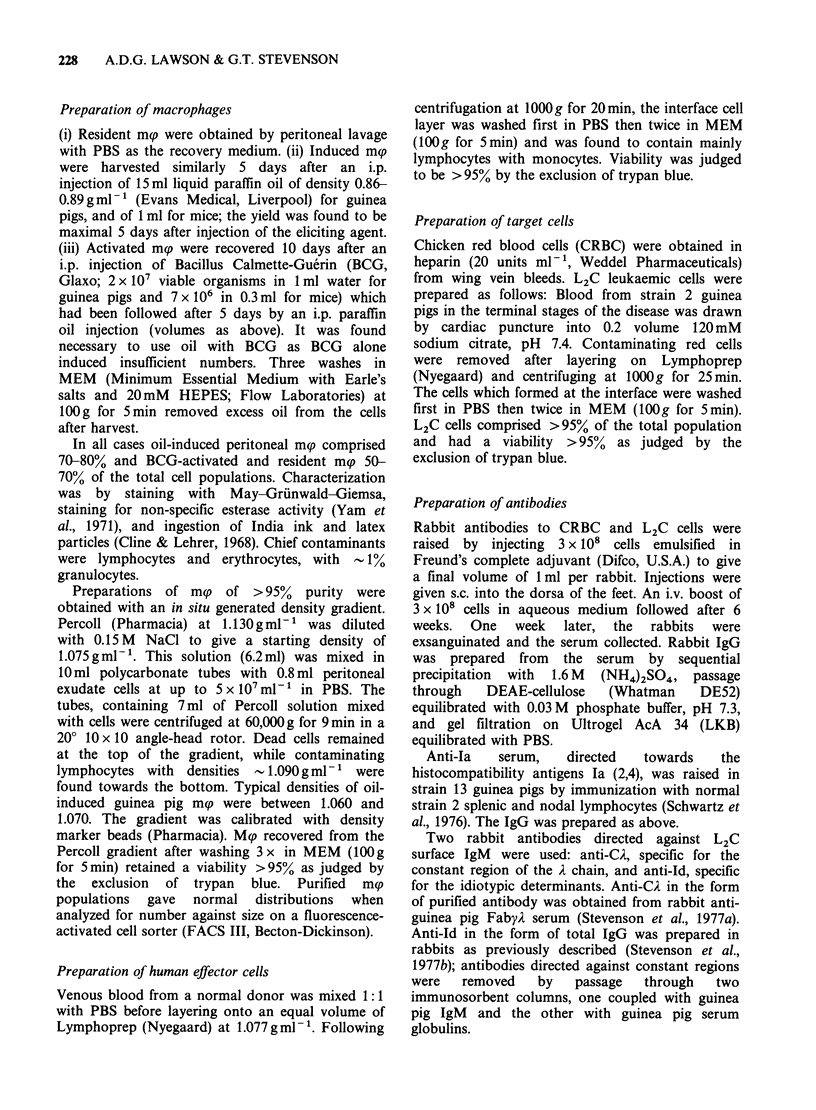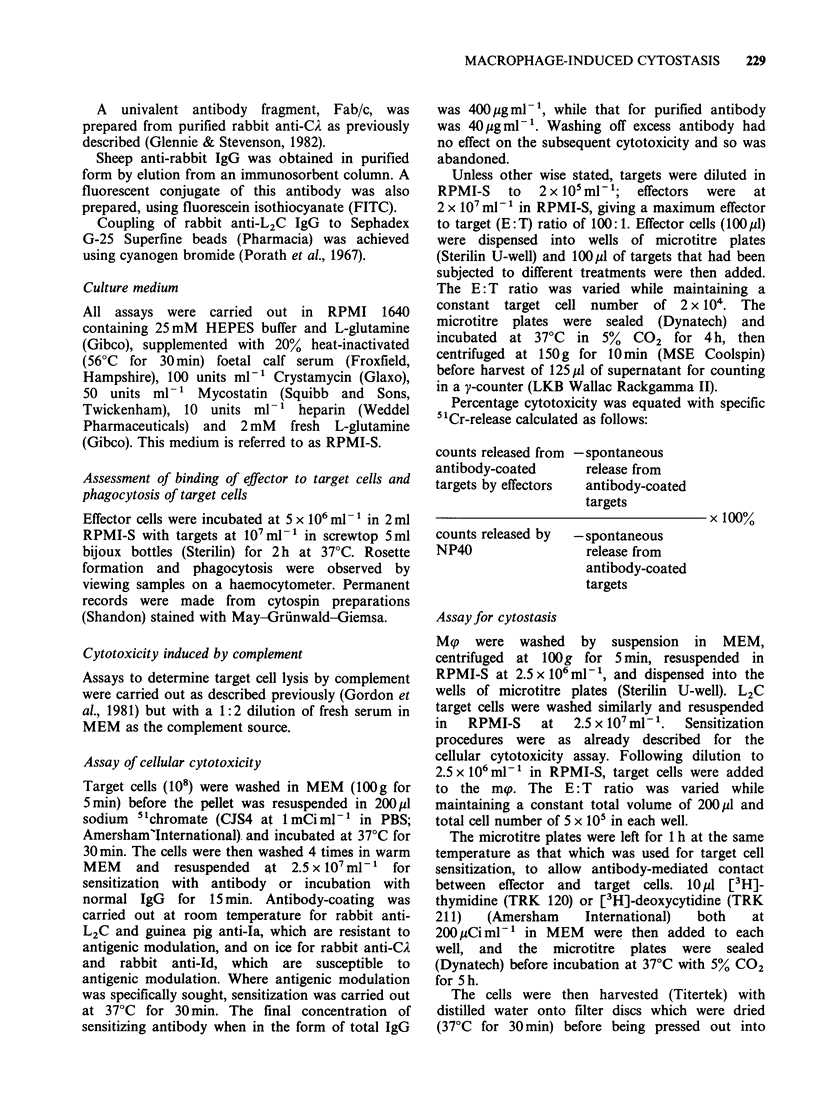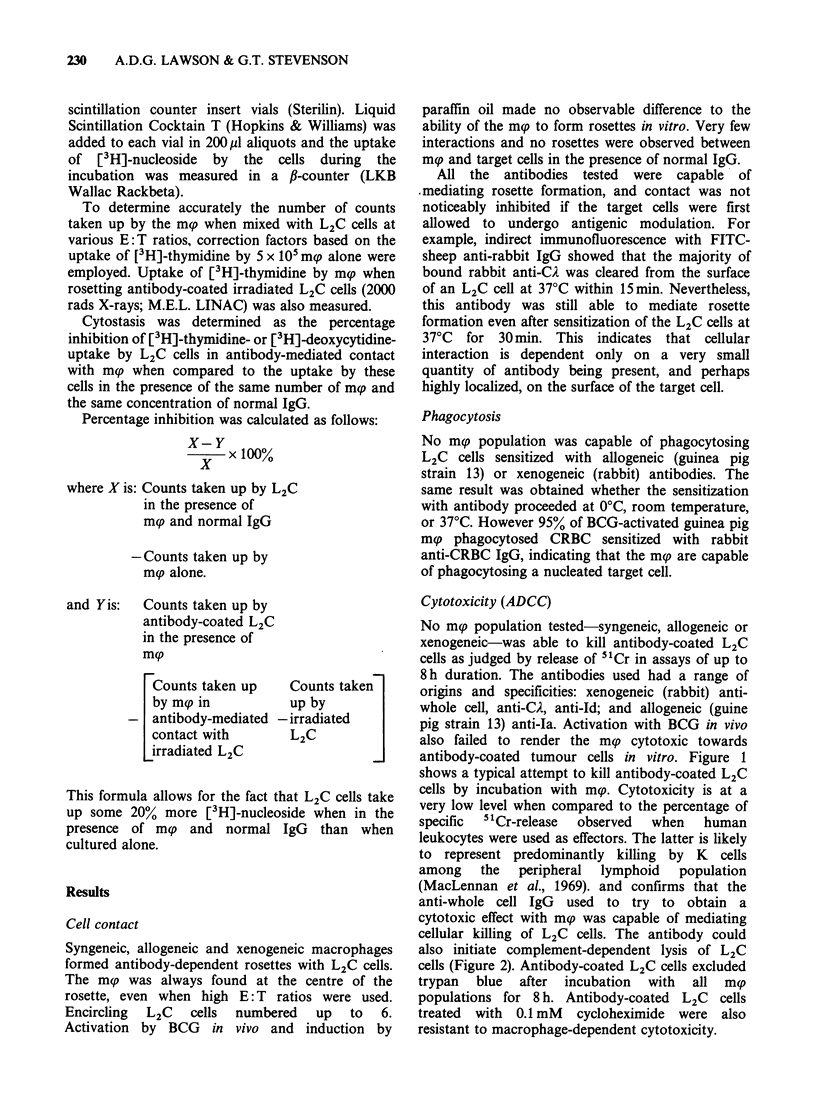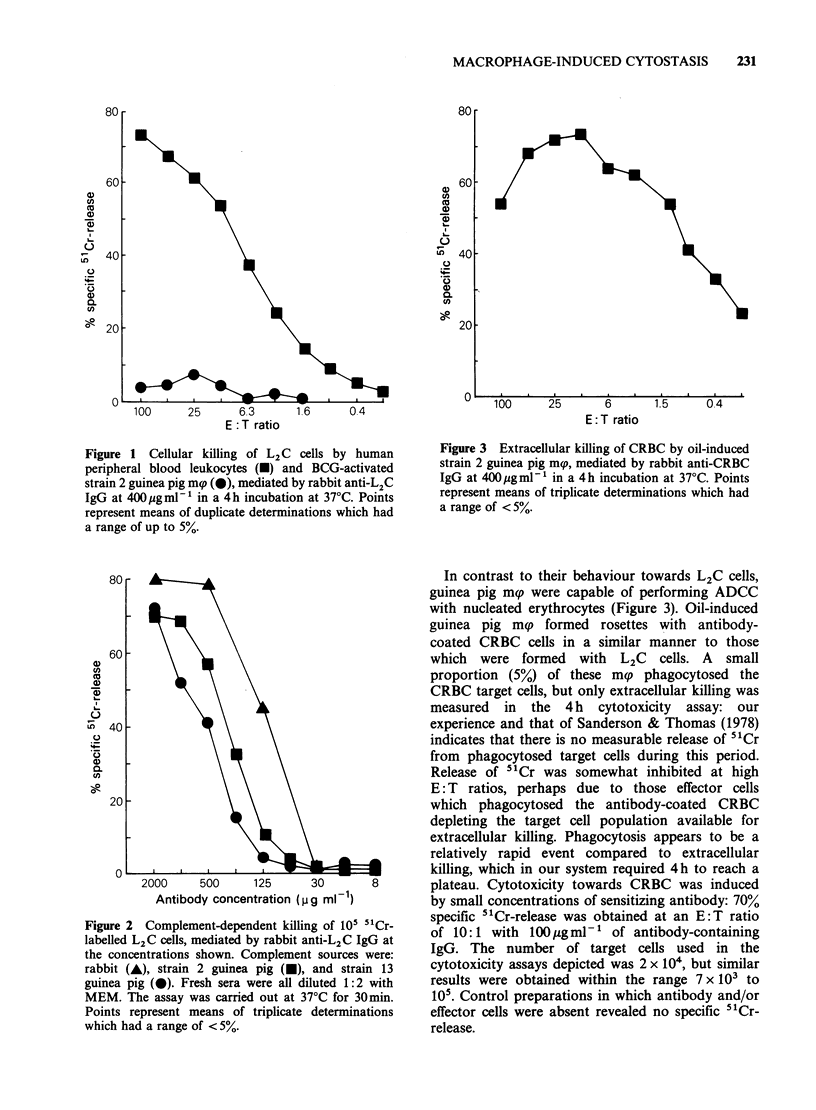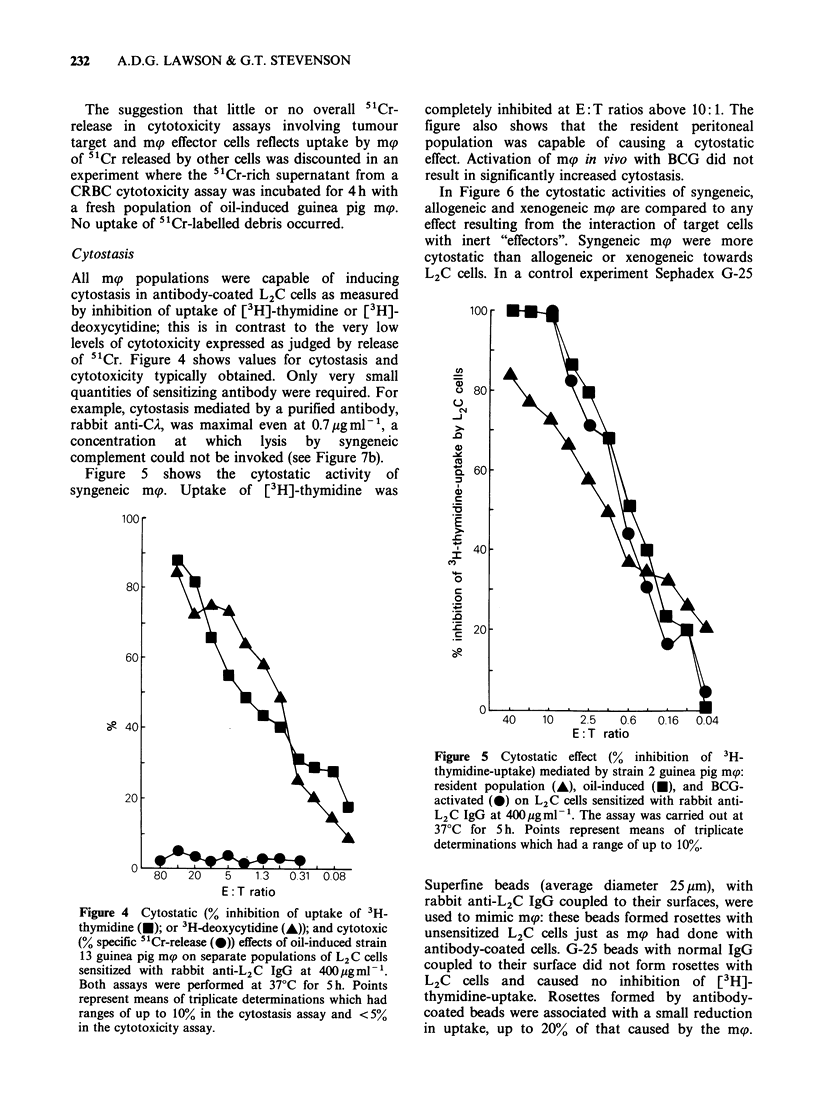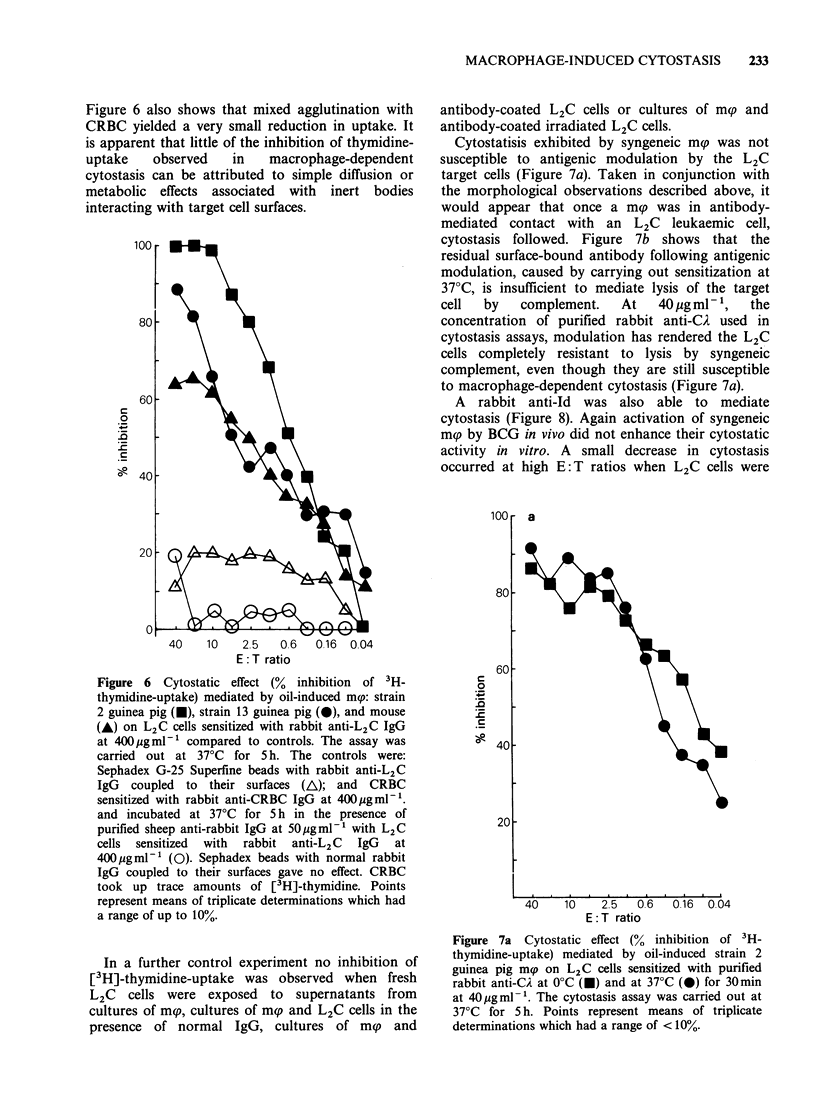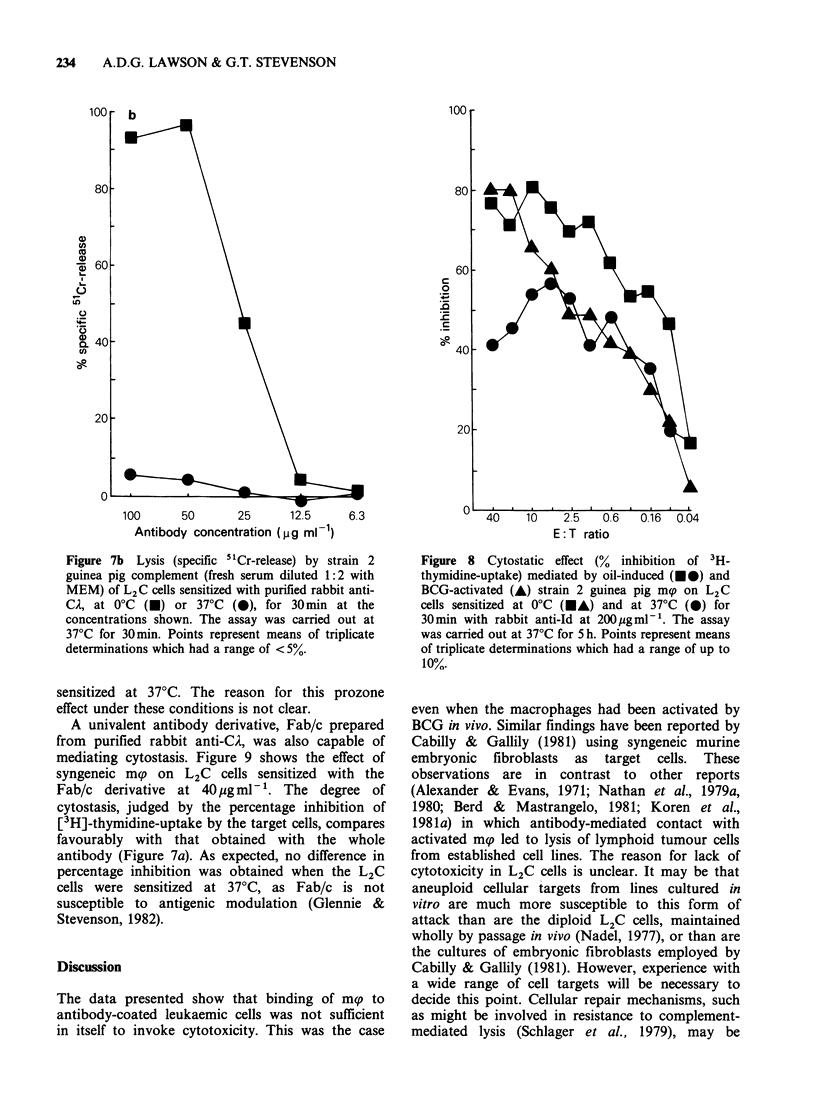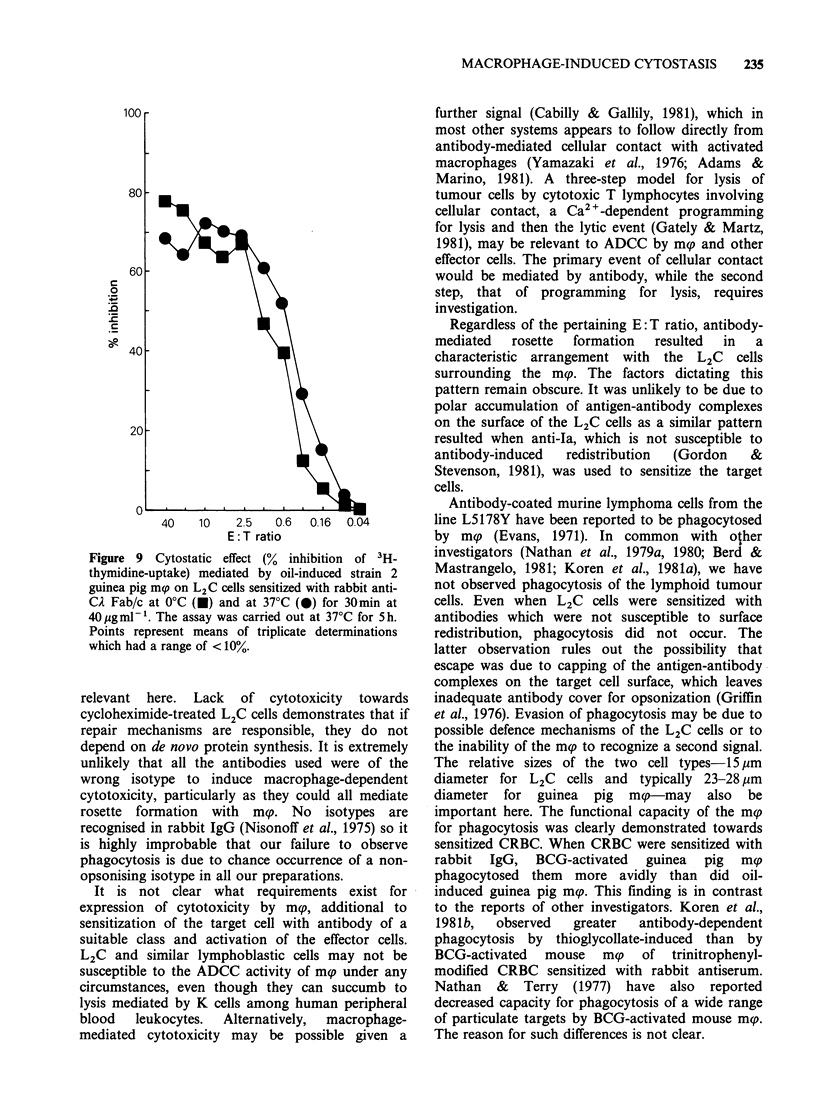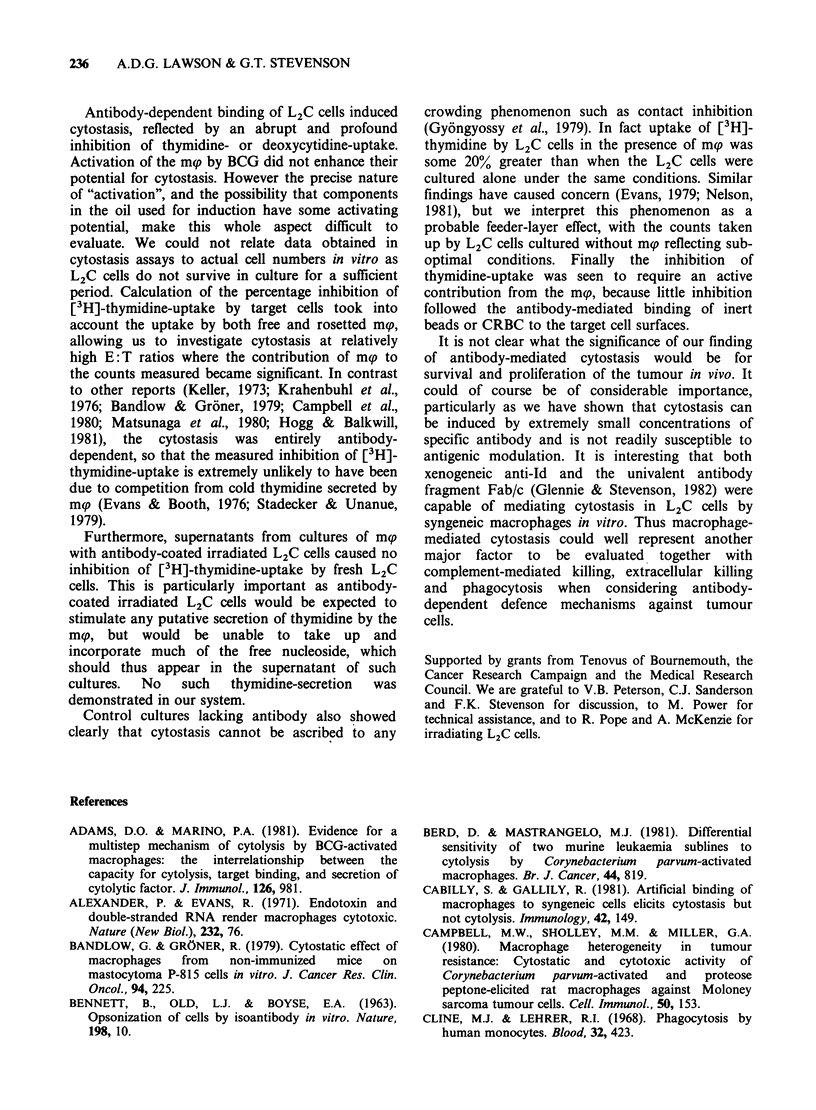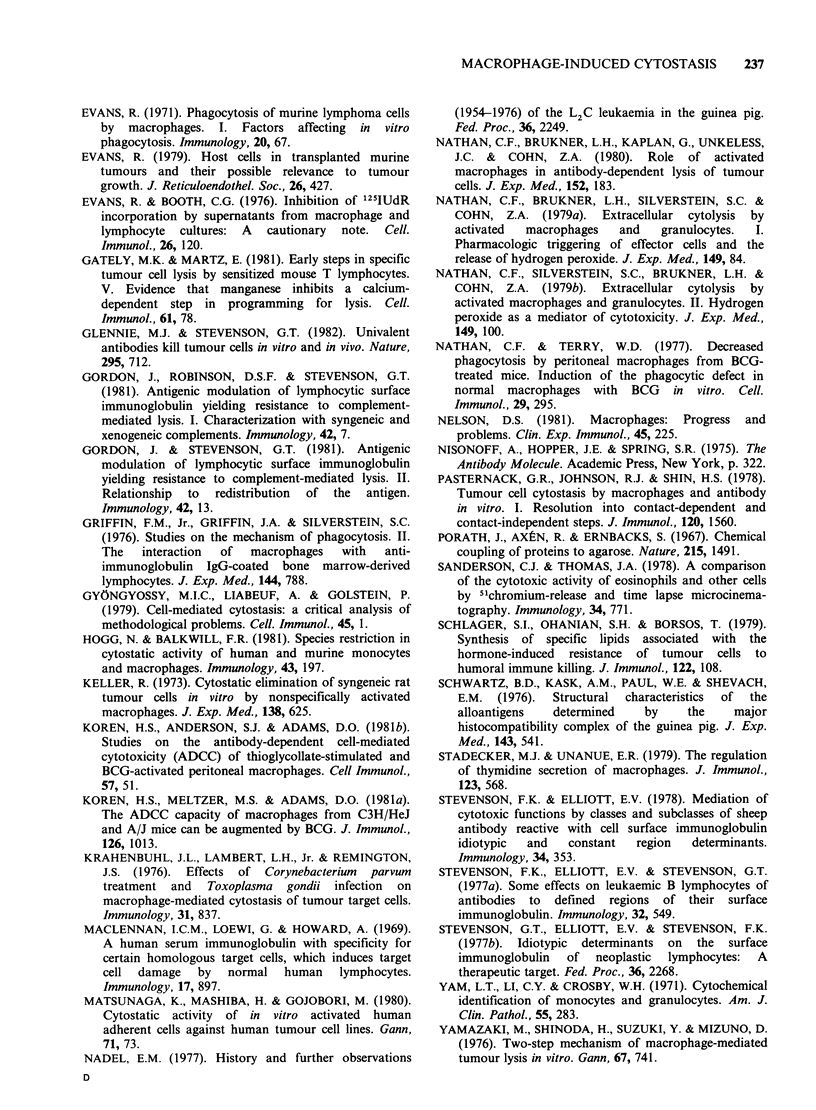# Macrophages induce antibody-dependent cytostasis but not lysis in guinea pig leukaemic cells.

**DOI:** 10.1038/bjc.1983.178

**Published:** 1983-08

**Authors:** A. D. Lawson, G. T. Stevenson

## Abstract

Guinea pig and mouse peritoneal macrophages formed antibody-dependent rosettes with guinea pig L2C leukaemic cells, but were unable either to phagocytose the cells or to kill them extracellularly as judged by the retention of 51Cr. Macrophages previously activated by BCG in vivo also failed to exhibit phagocytosis or cytoxicity towards the antibody-coated cells. These failures could not be attributed to deficient function of the macrophages nor to antigenic modulation of the L2C cells. The antibodies involved were capable of mediating lysis by complement, and ADCC by human leukocytes. However macrophages were cytostatic to antibody-coated L2C cells in that uptake of 3H-thymidine or 3H-deoxycytidine was abruptly and in some cases completely inhibited upon cell contact being established. Antigenic modulation which had proceeded sufficiently to protect against lysis by complement did not protect against cytostasis. Syngeneic macrophages had greater cytostatic activity than did allogeneic or xenogeneic. Macrophage activation by BCG did not result in significantly increased cytostasis. A univalent antibody derivative Fab/c was also capable of mediating cytostatis by the macrophages.


*Br. J. Cancer* (1983), **48**, 227–237

### Macrophages induce antibody-dependent cytostasis but not lysis in guinea pig leukaemic cells

A.D.G. Lawson & G.T. Stevenson

*Lymphoma Research Unit, Tenovus Research Laboratory, General Hospital, Southampton.*

**Summary** Guinea pig and mouse peritoneal macrophages formed antibody-dependent rosettes with guinea pig $L_2C$ leukaemic cells, but were unable either to phagocytose the cells or to kill them extracellularly as judged by the retention of $^{51}Cr$. Macrophages previously activated by BCG *in vivo* also failed to exhibit phagocytosis or cytoxicity towards the antibody-coated cells. These failures could not be attributed to deficient function of the macrophages nor to antigenic modulation of the $L_2C$ cells. The antibodies involved were capable of mediating lysis by complement, and ADCC by human leukocytes.

However macrophages were cytostatic to antibody-coated $L_2C$ cells in that uptake of $^3H$-thymidine or $^3H$-deoxycytidine was abruptly and in some cases completely inhibited upon cell contact being established. Antigenic modulation which had proceeded sufficiently to protect against lysis by complement did not protect against cytostasis. Syngeneic macrophages had greater cytostatic activity than did allogeneic or xenogeneic. Macrophage activation by BCG did not result in significantly increased cytostasis. A univalent antibody derivative Fab/c was also capable of mediating cytostatis by the macrophages.

Several reports that activated macrophages (m$\varphi$) are capable of mediating antibody-dependent cellular cytotoxicity (ADCC) towards lymphoid tumour cells (Alexander & Evans, 1971; Nathan *et al.*, 1979*a*, 1980; Berd & Mastrangelo, 1981; Koren *et al.*, 1981*a*) have prompted us to investigate their activity against neoplastic B lymphocytes of the guinea pig $L_2C$ leukaemia. Previous findings from this laboratory have demonstrated the susceptibility of these cells *in vitro* to antibody-dependent cytotoxicity—both extracellular killing by human peripheral blood leukocytes (Stevenson & Elliott, 1978) and complement-mediated lysis (Gordon *et al.*, 1981).

There are at least three mechanisms by which a m$\varphi$ can attack a tumour target cell. In phagocytosis, the m$\varphi$ ingests the target, presumably degrading it once it is internalized (Bennett *et al.*, 1963). In cytotoxicity, the m$\varphi$ lyses the target extracellularly, the mechanism possibly involving production of hydrogen peroxide (Nathan *et al.*, 1979*b*). Finally, a m$\varphi$ in antibody-mediated contact with a tumour target cell can inhibit its proliferative activity (Pasternack *et al.*, 1978). Such cell-mediated cytostasis must be distinguished from population phenomena such as contact inhibition (Gyöngyossy *et al.*, 1979).

In the present study syngeneic, allogeneic and xenogeneic m$\varphi$ were tested for their abilities to induce antibody-dependent cytotoxicity, cytostasis and phagocytosis of $L_2C$ cells *in vitro*. Lysis was assessed by the release of $^{51}Cr$, and cytostasis by inhibition of uptake of [$^3H$]-thymidine or [$^3H$]-deoxycytidine. M$\varphi$ populations were purified by density gradient centrifugation to reduce the possibility that any effects demonstrated were due to the presence of contaminating cells. In order to eliminate any artefacts arising from the use of heat-inactivated antiserum for sensitization, antibody-containing IgG or affinity-purified antibodies were used throughout. Xenogeneic anti-Id and the univalent antibody derivative Fab/c (Glennie & Stevenson, 1982) were used in some experiments to sensitize the $L_2C$ cells in an attempt to assess the possible significance of any effects determined *in vitro* for immunotherapy *in vivo*.

Our results show that cytostasis occurred in $L_2C$ cells following antibody-mediated contact with m$\varphi$, but neither phagocytosis nor cytotoxicity could be invoked even with the use of effector cells which had been activated by BCG *in vivo*. These results support the concept that antibody-dependent contact with the effector cell is a primary event, necessary but not sufficient for either phagocytosis or extracellular killing. A further requirement for phagocytosis, that the target cell be fully enveloped by antibody (Griffin *et al.*, 1976), also proved insufficient in our studies.

### Materials and methods

### *Animals*

New Zealand White rabbits, strain 2 and strain 13 guinea pigs and White Leghorn chickens were all bred on this site. Sheep and A strain mice were from Allington Farm, Porton, Wiltshire. Mature animals of either sex were used throughout.

Correspondence: G.T. Stevenson

Received 16 March 1983; accepted 21 April 1983.

© The Macmillan Press Ltd., 1983

*Preparation of macrophages*

(i) Resident m$\varphi$ were obtained by peritoneal lavage with PBS as the recovery medium. (ii) Induced m$\varphi$ were harvested similarly 5 days after an i.p. injection of 15 ml liquid paraffin oil of density 0.86–0.89 g ml$^{-1}$ (Evans Medical, Liverpool) for guinea pigs, and of 1 ml for mice; the yield was found to be maximal 5 days after injection of the eliciting agent. (iii) Activated m$\varphi$ were recovered 10 days after an i.p. injection of Bacillus Calmette-Guérin (BCG, Glaxo; $2 \times 10^7$ viable organisms in 1 ml water for guinea pigs and $7 \times 10^6$ in 0.3 ml for mice) which had been followed after 5 days by an i.p. paraffin oil injection (volumes as above). It was found necessary to use oil with BCG as BCG alone induced insufficient numbers. Three washes in MEM (Minimum Essential Medium with Earle's salts and 20 mM HEPES; Flow Laboratories) at 100 g for 5 min removed excess oil from the cells after harvest.

In all cases oil-induced peritoneal m$\varphi$ comprised 70–80% and BCG-activated and resident m$\varphi$ 50–70% of the total cell populations. Characterization was by staining with May–Grünwald–Giemsa, staining for non-specific esterase activity (Yam *et al.*, 1971), and ingestion of India ink and latex particles (Cline & Lehrer, 1968). Chief contaminants were lymphocytes and erythrocytes, with ~1% granulocytes.

Preparations of m$\varphi$ of >95% purity were obtained with an *in situ* generated density gradient. Percoll (Pharmacia) at 1.130 g ml$^{-1}$ was diluted with 0.15 M NaCl to give a starting density of 1.075 g ml$^{-1}$. This solution (6.2 ml) was mixed in 10 ml polycarbonate tubes with 0.8 ml peritoneal exudate cells at up to $5 \times 10^7$ ml$^{-1}$ in PBS. The tubes, containing 7 ml of Percoll solution mixed with cells were centrifuged at 60,000 g for 9 min in a 20° 10 × 10 angle-head rotor. Dead cells remained at the top of the gradient, while contaminating lymphocytes with densities ~1.090 g ml$^{-1}$ were found towards the bottom. Typical densities of oil-induced guinea pig m$\varphi$ were between 1.060 and 1.070. The gradient was calibrated with density marker beads (Pharmacia). M$\varphi$ recovered from the Percoll gradient after washing 3× in MEM (100 g for 5 min) retained a viability >95% as judged by the exclusion of trypan blue. Purified m$\varphi$ populations gave normal distributions when analyzed for number against size on a fluorescence-activated cell sorter (FACS III, Becton-Dickinson).

*Preparation of human effector cells*

Venous blood from a normal donor was mixed 1:1 with PBS before layering onto an equal volume of Lymphoprep (Nyegaard) at 1.077 g ml$^{-1}$. Following centrifugation at 1000 *g* for 20 min, the interface cell layer was washed first in PBS then twice in MEM (100 *g* for 5 min) and was found to contain mainly lymphocytes with monocytes. Viability was judged to be >95% by the exclusion of trypan blue.

*Preparation of target cells*

Chicken red blood cells (CRBC) were obtained in heparin (20 units ml$^{-1}$, Weddel Pharmaceuticals) from wing vein bleeds. $L_2C$ leukaemic cells were prepared as follows: Blood from strain 2 guinea pigs in the terminal stages of the disease was drawn by cardiac puncture into 0.2 volume 120 mM sodium citrate, pH 7.4. Contaminating red cells were removed after layering on Lymphoprep (Nyegaard) and centrifuging at 1000 *g* for 25 min. The cells which formed at the interface were washed first in PBS then twice in MEM (100 *g* for 5 min). $L_2C$ cells comprised >95% of the total population and had a viability >95% as judged by the exclusion of trypan blue.

*Preparation of antibodies*

Rabbit antibodies to CRBC and $L_2C$ cells were raised by injecting $3 \times 10^8$ cells emulsified in Freund's complete adjuvant (Difco, U.S.A.) to give a final volume of 1 ml per rabbit. Injections were given s.c. into the dorsa of the feet. An i.v. boost of $3 \times 10^8$ cells in aqueous medium followed after 6 weeks. One week later, the rabbits were exsanguinated and the serum collected. Rabbit IgG was prepared from the serum by sequential precipitation with 1.6 M $(NH_4)_2SO_4$, passage through DEAE-cellulose (Whatman DE52) equilibrated with 0.03 M phosphate buffer, pH 7.3, and gel filtration on Ultrogel AcA 34 (LKB) equilibrated with PBS.

Anti-Ia serum, directed towards the histocompatibility antigens Ia (2,4), was raised in strain 13 guinea pigs by immunization with normal strain 2 splenic and nodal lymphocytes (Schwartz *et al.*, 1976). The IgG was prepared as above.

Two rabbit antibodies directed against $L_2C$ surface IgM were used: anti-C$\lambda$, specific for the constant region of the $\lambda$ chain, and anti-Id, specific for the idiotypic determinants. Anti-C$\lambda$ in the form of purified antibody was obtained from rabbit anti-guinea pig Fab$\gamma\lambda$ serum (Stevenson *et al.*, 1977*a*). Anti-Id in the form of total IgG was prepared in rabbits as previously described (Stevenson *et al.*, 1977*b*); antibodies directed against constant regions were removed by passage through two immunosorbent columns, one coupled with guinea pig IgM and the other with guinea pig serum globulins.

A univalent antibody fragment, Fab/c, was prepared from purified rabbit anti-Cλ as previously described (Glennie & Stevenson, 1982).

Sheep anti-rabbit IgG was obtained in purified form by elution from an immunosorbent column. A fluorescent conjugate of this antibody was also prepared, using fluorescein isothiocyanate (FITC).

Coupling of rabbit anti-$L_2C$ IgG to Sephadex G-25 Superfine beads (Pharmacia) was achieved using cyanogen bromide (Porath *et al.*, 1967).

*Culture medium*

All assays were carried out in RPMI 1640 containing 25 mM HEPES buffer and L-glutamine (Gibco), supplemented with 20% heat-inactivated (56°C for 30 min) foetal calf serum (Froxfield, Hampshire), 100 units $ml^{-1}$ Crystamycin (Glaxo), 50 units $ml^{-1}$ Mycostatin (Squibb and Sons, Twickenham), 10 units $ml^{-1}$ heparin (Weddel Pharmaceuticals) and 2 mM fresh L-glutamine (Gibco). This medium is referred to as RPMI-S.

*Assessment of binding of effector to target cells and phagocytosis of target cells*

Effector cells were incubated at $5 \times 10^6$ $ml^{-1}$ in 2 ml RPMI-S with targets at $10^7$ $ml^{-1}$ in screwtop 5 ml bijoux bottles (Sterilin) for 2 h at 37°C. Rosette formation and phagocytosis were observed by viewing samples on a haemocytometer. Permanent records were made from cytospin preparations (Shandon) stained with May–Grünwald–Giemsa.

*Cytotoxicity induced by complement*

Assays to determine target cell lysis by complement were carried out as described previously (Gordon *et al.*, 1981) but with a 1:2 dilution of fresh serum in MEM as the complement source.

*Assay of cellular cytotoxicity*

Target cells ($10^8$) were washed in MEM (100 g for 5 min) before the pellet was resuspended in 200 μl sodium $^{51}$chromate (CJS4 at 1 mCi $ml^{-1}$ in PBS; Amersham International) and incubated at 37°C for 30 min. The cells were then washed 4 times in warm MEM and resuspended at $2.5 \times 10^7$ $ml^{-1}$ for sensitization with antibody or incubation with normal IgG for 15 min. Antibody-coating was carried out at room temperature for rabbit anti-$L_2C$ and guinea pig anti-Ia, which are resistant to antigenic modulation, and on ice for rabbit anti-Cλ and rabbit anti-Id, which are susceptible to antigenic modulation. Where antigenic modulation was specifically sought, sensitization was carried out at 37°C for 30 min. The final concentration of sensitizing antibody when in the form of total IgG was 400 μg $ml^{-1}$, while that for purified antibody was 40 μg $ml^{-1}$. Washing off excess antibody had no effect on the subsequent cytotoxicity and so was abandoned.

Unless other wise stated, targets were diluted in RPMI-S to $2 \times 10^5$ $ml^{-1}$; effectors were at $2 \times 10^7$ $ml^{-1}$ in RPMI-S, giving a maximum effector to target (E:T) ratio of 100:1. Effector cells (100 μl) were dispensed into wells of microtitre plates (Sterilin U-well) and 100 μl of targets that had been subjected to different treatments were then added. The E:T ratio was varied while maintaining a constant target cell number of $2 \times 10^4$. The microtitre plates were sealed (Dynatech) and incubated at 37°C in 5% $CO_2$ for 4 h, then centrifuged at 150 g for 10 min (MSE Coolspin) before harvest of 125 μl of supernatant for counting in a γ-counter (LKB Wallac Rackgamma II).

Percentage cytotoxicity was equated with specific $^{51}$Cr-release calculated as follows:

$$\frac{\text{counts released from antibody-coated targets by effectors} - \text{spontaneous release from antibody-coated targets}}{\text{counts released by NP40} - \text{spontaneous release from antibody-coated targets}} \times 100\%$$

*Assay for cytostasis*

Mφ were washed by suspension in MEM, centrifuged at 100 g for 5 min, resuspended in RPMI-S at $2.5 \times 10^6$ $ml^{-1}$, and dispensed into the wells of microtitre plates (Sterilin U-well). $L_2C$ target cells were washed similarly and resuspended in RPMI-S at $2.5 \times 10^7$ $ml^{-1}$. Sensitization procedures were as already described for the cellular cytotoxicity assay. Following dilution to $2.5 \times 10^6$ $ml^{-1}$ in RPMI-S, target cells were added to the mφ. The E:T ratio was varied while maintaining a constant total volume of 200 μl and total cell number of $5 \times 10^5$ in each well.

The microtitre plates were left for 1 h at the same temperature as that which was used for target cell sensitization, to allow antibody-mediated contact between effector and target cells. 10 μl [$^3$H]-thymidine (TRK 120) or [$^3$H]-deoxycytidine (TRK 211) (Amersham International) both at 200 μCi $ml^{-1}$ in MEM were then added to each well, and the microtitre plates were sealed (Dynatech) before incubation at 37°C with 5% $CO_2$ for 5 h.

The cells were then harvested (Titertek) with distilled water onto filter discs which were dried (37°C for 30 min) before being pressed out into scintillation counter insert vials (Sterilin). Liquid Scintillation Cocktain T (Hopkins & Williams) was added to each vial in 200 $\mu$l aliquots and the uptake of [$^3$H]-nucleoside by the cells during the incubation was measured in a $\beta$-counter (LKB Wallac Rackbeta).

To determine accurately the number of counts taken up by the m$\varphi$ when mixed with $L_2C$ cells at various E:T ratios, correction factors based on the uptake of [$^3$H]-thymidine by $5 \times 10^5$ m$\varphi$ alone were employed. Uptake of [$^3$H]-thymidine by m$\varphi$ when rosetting antibody-coated irradiated $L_2C$ cells (2000 rads X-rays; M.E.L. LINAC) was also measured.

Cytostasis was determined as the percentage inhibition of [$^3$H]-thymidine- or [$^3$H]-deoxycytidine-uptake by $L_2C$ cells in antibody-mediated contact with m$\varphi$ when compared to the uptake by these cells in the presence of the same number of m$\varphi$ and the same concentration of normal IgG.

Percentage inhibition was calculated as follows:

$$\frac{X-Y}{X} \times 100\%$$

where $X$ is: Counts taken up by $L_2C$ in the presence of m$\varphi$ and normal IgG

− Counts taken up by m$\varphi$ alone.

and $Y$ is: Counts taken up by antibody-coated $L_2C$ in the presence of m$\varphi$

− [Counts taken up by m$\varphi$ in antibody-mediated contact with irradiated $L_2C$ − Counts taken up by irradiated $L_2C$]

This formula allows for the fact that $L_2C$ cells take up some 20% more [$^3$H]-nucleoside when in the presence of m$\varphi$ and normal IgG than when cultured alone.

### Results

### *Cell contact*

Syngeneic, allogeneic and xenogeneic macrophages formed antibody-dependent rosettes with $L_2C$ cells. The m$\varphi$ was always found at the centre of the rosette, even when high E:T ratios were used. Encircling $L_2C$ cells numbered up to 6. Activation by BCG *in vivo* and induction by paraffin oil made no observable difference to the ability of the m$\varphi$ to form rosettes *in vitro*. Very few interactions and no rosettes were observed between m$\varphi$ and target cells in the presence of normal IgG.

All the antibodies tested were capable of mediating rosette formation, and contact was not noticeably inhibited if the target cells were first allowed to undergo antigenic modulation. For example, indirect immunofluorescence with FITC-sheep anti-rabbit IgG showed that the majority of bound rabbit anti-C$\lambda$ was cleared from the surface of an $L_2C$ cell at 37°C within 15 min. Nevertheless, this antibody was still able to mediate rosette formation even after sensitization of the $L_2C$ cells at 37°C for 30 min. This indicates that cellular interaction is dependent only on a very small quantity of antibody being present, and perhaps highly localized, on the surface of the target cell.

### *Phagocytosis*

No m$\varphi$ population was capable of phagocytosing $L_2C$ cells sensitized with allogeneic (guinea pig strain 13) or xenogeneic (rabbit) antibodies. The same result was obtained whether the sensitization with antibody proceeded at 0°C, room temperature, or 37°C. However 95% of BCG-activated guinea pig m$\varphi$ phagocytosed CRBC sensitized with rabbit anti-CRBC IgG, indicating that the m$\varphi$ are capable of phagocytosing a nucleated target cell.

### *Cytotoxicity (ADCC)*

No m$\varphi$ population tested—syngeneic, allogeneic or xenogeneic—was able to kill antibody-coated $L_2C$ cells as judged by release of $^{51}$Cr in assays of up to 8 h duration. The antibodies used had a range of origins and specificities: xenogeneic (rabbit) anti-whole cell, anti-C$\lambda$, anti-Id; and allogeneic (guine pig strain 13) anti-Ia. Activation with BCG *in vivo* also failed to render the m$\varphi$ cytotoxic towards antibody-coated tumour cells *in vitro*. Figure 1 shows a typical attempt to kill antibody-coated $L_2C$ cells by incubation with m$\varphi$. Cytotoxicity is at a very low level when compared to the percentage of specific $^{51}$Cr-release observed when human leukocytes were used as effectors. The latter is likely to represent predominantly killing by K cells among the peripheral lymphoid population (MacLennan *et al.*, 1969). and confirms that the anti-whole cell IgG used to try to obtain a cytotoxic effect with m$\varphi$ was capable of mediating cellular killing of $L_2C$ cells. The antibody could also initiate complement-dependent lysis of $L_2C$ cells (Figure 2). Antibody-coated $L_2C$ cells excluded trypan blue after incubation with all m$\varphi$ populations for 8 h. Antibody-coated $L_2C$ cells treated with 0.1 mM cycloheximide were also resistant to macrophage-dependent cytotoxicity.

**Figure 1** Cellular killing of $L_2C$ cells by human peripheral blood leukocytes (■) and BCG-activated strain 2 guinea pig m$\varphi$ (●), mediated by rabbit anti-$L_2C$ IgG at 400 $\mu g\,ml^{-1}$ in a 4 h incubation at 37°C. Points represent means of duplicate determinations which had a range of up to 5%.

**Figure 3** Extracellular killing of CRBC by oil-induced strain 2 guinea pig m$\varphi$, mediated by rabbit anti-CRBC IgG at 400 $\mu g\,ml^{-1}$ in a 4 h incubation at 37°C. Points represent means of triplicate determinations which had a range of < 5%.

**Figure 2** Complement-dependent killing of $10^5$ $^{51}$Cr-labelled $L_2C$ cells, mediated by rabbit anti-$L_2C$ IgG at the concentrations shown. Complement sources were: rabbit (▲), strain 2 guinea pig (■), and strain 13 guinea pig (●). Fresh sera were all diluted 1:2 with MEM. The assay was carried out at 37°C for 30 min. Points represent means of triplicate determinations which had a range of < 5%.

In contrast to their behaviour towards $L_2C$ cells, guinea pig m$\varphi$ were capable of performing ADCC with nucleated erythrocytes (Figure 3). Oil-induced guinea pig m$\varphi$ formed rosettes with antibody-coated CRBC cells in a similar manner to those which were formed with $L_2C$ cells. A small proportion (5%) of these m$\varphi$ phagocytosed the CRBC target cells, but only extracellular killing was measured in the 4 h cytotoxicity assay: our experience and that of Sanderson & Thomas (1978) indicates that there is no measurable release of $^{51}$Cr from phagocytosed target cells during this period. Release of $^{51}$Cr was somewhat inhibited at high E:T ratios, perhaps due to those effector cells which phagocytosed the antibody-coated CRBC depleting the target cell population available for extracellular killing. Phagocytosis appears to be a relatively rapid event compared to extracellular killing, which in our system required 4 h to reach a plateau. Cytotoxicity towards CRBC was induced by small concentrations of sensitizing antibody: 70% specific $^{51}$Cr-release was obtained at an E:T ratio of 10:1 with 100 $\mu g\,ml^{-1}$ of antibody-containing IgG. The number of target cells used in the cytotoxicity assays depicted was $2 \times 10^4$, but similar results were obtained within the range $7 \times 10^3$ to $10^5$. Control preparations in which antibody and/or effector cells were absent revealed no specific $^{51}$Cr-release.

The suggestion that little or no overall $^{51}Cr$-release in cytotoxicity assays involving tumour target and m$\varphi$ effector cells reflects uptake by m$\varphi$ of $^{51}Cr$ released by other cells was discounted in an experiment where the $^{51}Cr$-rich supernatant from a CRBC cytotoxicity assay was incubated for 4 h with a fresh population of oil-induced guinea pig m$\varphi$. No uptake of $^{51}Cr$-labelled debris occurred.

### *Cytostasis*

All m$\varphi$ populations were capable of inducing cytostasis in antibody-coated $L_2C$ cells as measured by inhibition of uptake of [$^3$H]-thymidine or [$^3$H]-deoxycytidine; this is in contrast to the very low levels of cytotoxicity expressed as judged by release of $^{51}Cr$. Figure 4 shows values for cytostasis and cytotoxicity typically obtained. Only very small quantities of sensitizing antibody were required. For example, cytostasis mediated by a purified antibody, rabbit anti-C$\lambda$, was maximal even at 0.7 $\mu g\,ml^{-1}$, a concentration at which lysis by syngeneic complement could not be invoked (see Figure 7b).

Figure 5 shows the cytostatic activity of syngeneic m$\varphi$. Uptake of [$^3$H]-thymidine was completely inhibited at E:T ratios above 10:1. The figure also shows that the resident peritoneal population was capable of causing a cytostatic effect. Activation of m$\varphi$ *in vivo* with BCG did not result in significantly increased cytostasis.

In Figure 6 the cytostatic activities of syngeneic, allogeneic and xenogeneic m$\varphi$ are compared to any effect resulting from the interaction of target cells with inert "effectors". Syngeneic m$\varphi$ were more cytostatic than allogeneic or xenogeneic towards $L_2C$ cells. In a control experiment Sephadex G-25

**Figure 4** Cytostatic (% inhibition of uptake of $^3$H-thymidine (■); or $^3$H-deoxycytidine (▲)); and cytotoxic (% specific $^{51}$Cr-release (●)) effects of oil-induced strain 13 guinea pig m$\varphi$ on separate populations of $L_2C$ cells sensitized with rabbit anti-$L_2C$ IgG at 400 $\mu g\,ml^{-1}$. Both assays were performed at 37°C for 5 h. Points represent means of triplicate determinations which had ranges of up to 10% in the cytostasis assay and <5% in the cytotoxicity assay.

**Figure 5** Cytostatic effect (% inhibition of $^3$H-thymidine-uptake) mediated by strain 2 guinea pig m$\varphi$: resident population (▲), oil-induced (■), and BCG-activated (●) on $L_2C$ cells sensitized with rabbit anti-$L_2C$ IgG at 400 $\mu g\,ml^{-1}$. The assay was carried out at 37°C for 5 h. Points represent means of triplicate determinations which had a range of up to 10%.

Superfine beads (average diameter 25 $\mu$m), with rabbit anti-$L_2C$ IgG coupled to their surfaces, were used to mimic m$\varphi$: these beads formed rosettes with unsensitized $L_2C$ cells just as m$\varphi$ had done with antibody-coated cells. G-25 beads with normal IgG coupled to their surface did not form rosettes with $L_2C$ cells and caused no inhibition of [$^3$H]-thymidine-uptake. Rosettes formed by antibody-coated beads were associated with a small reduction in uptake, up to 20% of that caused by the m$\varphi$.

Figure 6 also shows that mixed agglutination with CRBC yielded a very small reduction in uptake. It is apparent that little of the inhibition of thymidine-uptake observed in macrophage-dependent cytostasis can be attributed to simple diffusion or metabolic effects associated with inert bodies interacting with target cell surfaces.

**Figure 6** Cytostatic effect (% inhibition of $^3$H-thymidine-uptake) mediated by oil-induced m$\varphi$: strain 2 guinea pig (■), strain 13 guinea pig (●), and mouse (▲) on $L_2C$ cells sensitized with rabbit anti-$L_2C$ IgG at $400 \mu g\, ml^{-1}$ compared to controls. The assay was carried out at 37°C for 5 h. The controls were: Sephadex G-25 Superfine beads with rabbit anti-$L_2C$ IgG coupled to their surfaces (△); and CRBC sensitized with rabbit anti-CRBC IgG at $400 \mu g\, ml^{-1}$. and incubated at 37°C for 5 h in the presence of purified sheep anti-rabbit IgG at $50 \mu g\, ml^{-1}$ with $L_2C$ cells sensitized with rabbit anti-$L_2C$ IgG at $400 \mu g\, ml^{-1}$ (○). Sephadex beads with normal rabbit IgG coupled to their surfaces gave no effect. CRBC took up trace amounts of [$^3$H]-thymidine. Points represent means of triplicate determinations which had a range of up to 10%.

In a further control experiment no inhibition of [$^3$H]-thymidine-uptake was observed when fresh $L_2C$ cells were exposed to supernatants from cultures of m$\varphi$, cultures of m$\varphi$ and $L_2C$ cells in the presence of normal IgG, cultures of m$\varphi$ and antibody-coated $L_2C$ cells or cultures of m$\varphi$ and antibody-coated irradiated $L_2C$ cells.

Cytostatisis exhibited by syngeneic m$\varphi$ was not susceptible to antigenic modulation by the $L_2C$ target cells (Figure 7*a*). Taken in conjunction with the morphological observations described above, it would appear that once a m$\varphi$ was in antibody-mediated contact with an $L_2C$ leukaemic cell, cytostasis followed. Figure 7*b* shows that the residual surface-bound antibody following antigenic modulation, caused by carrying out sensitization at 37°C, is insufficient to mediate lysis of the target cell by complement. At $40 \mu g\, ml^{-1}$, the concentration of purified rabbit anti-C$\lambda$ used in cytostasis assays, modulation has rendered the $L_2C$ cells completely resistant to lysis by syngeneic complement, even though they are still susceptible to macrophage-dependent cytostasis (Figure 7*a*).

A rabbit anti-Id was also able to mediate cytostasis (Figure 8). Again activation of syngeneic m$\varphi$ by BCG *in vivo* did not enhance their cytostatic activity *in vitro*. A small decrease in cytostasis occurred at high E:T ratios when $L_2C$ cells were

**Figure 7a** Cytostatic effect (% inhibition of $^3$H-thymidine-uptake) mediated by oil-induced strain 2 guinea pig m$\varphi$ on $L_2C$ cells sensitized with purified rabbit anti-C$\lambda$ at 0°C (■) and at 37°C (●) for 30 min at $40 \mu g\, ml^{-1}$. The cytostasis assay was carried out at 37°C for 5 h. Points represent means of triplicate determinations which had a range of $<10\%$.

**Figure 7b** Lysis (specific $^{51}$Cr-release) by strain 2 guinea pig complement (fresh serum diluted 1:2 with MEM) of $L_2C$ cells sensitized with purified rabbit anti-Cλ, at 0°C (■) or 37°C (●), for 30 min at the concentrations shown. The assay was carried out at 37°C for 30 min. Points represent means of triplicate determinations which had a range of <5%.

**Figure 8** Cytostatic effect (% inhibition of $^3$H-thymidine-uptake) mediated by oil-induced (■●) and BCG-activated (▲) strain 2 guinea pig mφ on $L_2C$ cells sensitized at 0°C (■▲) and at 37°C (●) for 30 min with rabbit anti-Id at 200 μg ml$^{-1}$. The assay was carried out at 37°C for 5 h. Points represent means of triplicate determinations which had a range of up to 10%.

sensitized at 37°C. The reason for this prozone effect under these conditions is not clear.

A univalent antibody derivative, Fab/c prepared from purified rabbit anti-Cλ, was also capable of mediating cytostasis. Figure 9 shows the effect of syngeneic mφ on $L_2C$ cells sensitized with the Fab/c derivative at 40 μg ml$^{-1}$. The degree of cytostasis, judged by the percentage inhibition of [$^3$H]-thymidine-uptake by the target cells, compares favourably with that obtained with the whole antibody (Figure 7*a*). As expected, no difference in percentage inhibition was obtained when the $L_2C$ cells were sensitized at 37°C, as Fab/c is not susceptible to antigenic modulation (Glennie & Stevenson, 1982).

### Discussion

The data presented show that binding of mφ to antibody-coated leukaemic cells was not sufficient in itself to invoke cytotoxicity. This was the case even when the macrophages had been activated by BCG *in vivo*. Similar findings have been reported by Cabilly & Gallily (1981) using syngeneic murine embryonic fibroblasts as target cells. These observations are in contrast to other reports (Alexander & Evans, 1971; Nathan *et al.*, 1979*a*, 1980; Berd & Mastrangelo, 1981; Koren *et al.*, 1981*a*) in which antibody-mediated contact with activated mφ led to lysis of lymphoid tumour cells from established cell lines. The reason for lack of cytotoxicity in $L_2C$ cells is unclear. It may be that aneuploid cellular targets from lines cultured *in vitro* are much more susceptible to this form of attack than are the diploid $L_2C$ cells, maintained wholly by passage *in vivo* (Nadel, 1977), or than are the cultures of embryonic fibroblasts employed by Cabilly & Gallily (1981). However, experience with a wide range of cell targets will be necessary to decide this point. Cellular repair mechanisms, such as might be involved in resistance to complement-mediated lysis (Schlager *et al.*, 1979), may be

**Figure 9** Cytostatic effect (% inhibition of $^3$H-thymidine-uptake) mediated by oil-induced strain 2 guinea pig m$\varphi$ on $L_2C$ cells sensitized with rabbit anti-Cλ Fab/c at 0°C (■) and at 37°C (●) for 30 min at 40 $\mu$g ml$^{-1}$. The assay was carried out at 37°C for 5 h. Points represent means of triplicate determinations which had a range of <10%.

relevant here. Lack of cytotoxicity towards cycloheximide-treated $L_2C$ cells demonstrates that if repair mechanisms are responsible, they do not depend on *de novo* protein synthesis. It is extremely unlikely that all the antibodies used were of the wrong isotype to induce macrophage-dependent cytotoxicity, particularly as they could all mediate rosette formation with m$\varphi$. No isotypes are recognised in rabbit IgG (Nisonoff *et al.*, 1975) so it is highly improbable that our failure to observe phagocytosis is due to chance occurrence of a non-opsonising isotype in all our preparations.

It is not clear what requirements exist for expression of cytotoxicity by m$\varphi$, additional to sensitization of the target cell with antibody of a suitable class and activation of the effector cells. $L_2C$ and similar lymphoblastic cells may not be susceptible to the ADCC activity of m$\varphi$ under any circumstances, even though they can succumb to lysis mediated by K cells among human peripheral blood leukocytes. Alternatively, macrophage-mediated cytotoxicity may be possible given a further signal (Cabilly & Gallily, 1981), which in most other systems appears to follow directly from antibody-mediated cellular contact with activated macrophages (Yamazaki *et al.*, 1976; Adams & Marino, 1981). A three-step model for lysis of tumour cells by cytotoxic T lymphocytes involving cellular contact, a $Ca^{2+}$-dependent programming for lysis and then the lytic event (Gately & Martz, 1981), may be relevant to ADCC by m$\varphi$ and other effector cells. The primary event of cellular contact would be mediated by antibody, while the second step, that of programming for lysis, requires investigation.

Regardless of the pertaining E:T ratio, antibody-mediated rosette formation resulted in a characteristic arrangement with the $L_2C$ cells surrounding the m$\varphi$. The factors dictating this pattern remain obscure. It was unlikely to be due to polar accumulation of antigen-antibody complexes on the surface of the $L_2C$ cells as a similar pattern resulted when anti-Ia, which is not susceptible to antibody-induced redistribution (Gordon & Stevenson, 1981), was used to sensitize the target cells.

Antibody-coated murine lymphoma cells from the line L5178Y have been reported to be phagocytosed by m$\varphi$ (Evans, 1971). In common with other investigators (Nathan *et al.*, 1979*a*, 1980; Berd & Mastrangelo, 1981; Koren *et al.*, 1981*a*), we have not observed phagocytosis of the lymphoid tumour cells. Even when $L_2C$ cells were sensitized with antibodies which were not susceptible to surface redistribution, phagocytosis did not occur. The latter observation rules out the possibility that escape was due to capping of the antigen-antibody complexes on the target cell surface, which leaves inadequate antibody cover for opsonization (Griffin *et al.*, 1976). Evasion of phagocytosis may be due to possible defence mechanisms of the $L_2C$ cells or to the inability of the m$\varphi$ to recognize a second signal. The relative sizes of the two cell types—15 $\mu$m diameter for $L_2C$ cells and typically 23–28 $\mu$m diameter for guinea pig m$\varphi$—may also be important here. The functional capacity of the m$\varphi$ for phagocytosis was clearly demonstrated towards sensitized CRBC. When CRBC were sensitized with rabbit IgG, BCG-activated guinea pig m$\varphi$ phagocytosed them more avidly than did oil-induced guinea pig m$\varphi$. This finding is in contrast to the reports of other investigators. Koren *et al.*, 1981*b*, observed greater antibody-dependent phagocytosis by thioglycollate-induced than by BCG-activated mouse m$\varphi$ of trinitrophenyl-modified CRBC sensitized with rabbit antiserum. Nathan & Terry (1977) have also reported decreased capacity for phagocytosis of a wide range of particulate targets by BCG-activated mouse m$\varphi$. The reason for such differences is not clear.

Antibody-dependent binding of $L_2C$ cells induced cytostasis, reflected by an abrupt and profound inhibition of thymidine- or deoxycytidine-uptake. Activation of the m$\varphi$ by BCG did not enhance their potential for cytostasis. However the precise nature of "activation", and the possibility that components in the oil used for induction have some activating potential, make this whole aspect difficult to evaluate. We could not relate data obtained in cytostasis assays to actual cell numbers *in vitro* as $L_2C$ cells do not survive in culture for a sufficient period. Calculation of the percentage inhibition of [$^3$H]-thymidine-uptake by target cells took into account the uptake by both free and rosetted m$\varphi$, allowing us to investigate cytostasis at relatively high E:T ratios where the contribution of m$\varphi$ to the counts measured became significant. In contrast to other reports (Keller, 1973; Krahenbuhl *et al.*, 1976; Bandlow & Gröner, 1979; Campbell *et al.*, 1980; Matsunaga *et al.*, 1980; Hogg & Balkwill, 1981), the cytostasis was entirely antibody-dependent, so that the measured inhibition of [$^3$H]-thymidine-uptake is extremely unlikely to have been due to competition from cold thymidine secreted by m$\varphi$ (Evans & Booth, 1976; Stadecker & Unanue, 1979).

Furthermore, supernatants from cultures of m$\varphi$ with antibody-coated irradiated $L_2C$ cells caused no inhibition of [$^3$H]-thymidine-uptake by fresh $L_2C$ cells. This is particularly important as antibody-coated irradiated $L_2C$ cells would be expected to stimulate any putative secretion of thymidine by the m$\varphi$, but would be unable to take up and incorporate much of the free nucleoside, which should thus appear in the supernatant of such cultures. No such thymidine-secretion was demonstrated in our system.

Control cultures lacking antibody also showed clearly that cytostasis cannot be ascribed to any crowding phenomenon such as contact inhibition (Gyöngyossy *et al.*, 1979). In fact uptake of [$^3$H]-thymidine by $L_2C$ cells in the presence of m$\varphi$ was some 20% greater than when the $L_2C$ cells were cultured alone under the same conditions. Similar findings have caused concern (Evans, 1979; Nelson, 1981), but we interpret this phenomenon as a probable feeder-layer effect, with the counts taken up by $L_2C$ cells cultured without m$\varphi$ reflecting sub-optimal conditions. Finally the inhibition of thymidine-uptake was seen to require an active contribution from the m$\varphi$, because little inhibition followed the antibody-mediated binding of inert beads or CRBC to the target cell surfaces.

It is not clear what the significance of our finding of antibody-mediated cytostasis would be for survival and proliferation of the tumour *in vivo*. It could of course be of considerable importance, particularly as we have shown that cytostasis can be induced by extremely small concentrations of specific antibody and is not readily susceptible to antigenic modulation. It is interesting that both xenogeneic anti-Id and the univalent antibody fragment Fab/c (Glennie & Stevenson, 1982) were capable of mediating cytostasis in $L_2C$ cells by syngeneic macrophages *in vitro*. Thus macrophage-mediated cytostasis could well represent another major factor to be evaluated together with complement-mediated killing, extracellular killing and phagocytosis when considering antibody-dependent defence mechanisms against tumour cells.

Supported by grants from Tenovus of Bournemouth, the Cancer Research Campaign and the Medical Research Council. We are grateful to V.B. Peterson, C.J. Sanderson and F.K. Stevenson for discussion, to M. Power for technical assistance, and to R. Pope and A. McKenzie for irradiating $L_2C$ cells.

### References

ADAMS, D.O. & MARINO, P.A. (1981). Evidence for a multistep mechanism of cytolysis by BCG-activated macrophages: the interrelationship between the capacity for cytolysis, target binding, and secretion of cytolytic factor. *J. Immunol.*, **126**, 981.

ALEXANDER, P. & EVANS, R. (1971). Endotoxin and double-stranded RNA render macrophages cytotoxic. *Nature* (*New Biol.*), **232**, 76.

BANDLOW, G. & GRÖNER, R. (1979). Cytostatic effect of macrophages from non-immunized mice on mastocytoma P-815 cells *in vitro*. *J. Cancer Res. Clin. Oncol.*, **94**, 225.

BENNETT, B., OLD, L.J. & BOYSE, E.A. (1963). Opsonization of cells by isoantibody *in vitro*. *Nature*, **198**, 10.

BERD, D. & MASTRANGELO, M.J. (1981). Differential sensitivity of two murine leukaemia sublines to cytolysis by *Corynebacterium parvum*-activated macrophages. *Br. J. Cancer*, **44**, 819.

CABILLY, S. & GALLILY, R. (1981). Artificial binding of macrophages to syngeneic cells elicits cytostasis but not cytolysis. *Immunology*, **42**, 149.

CAMPBELL, M.W., SHOLLEY, M.M. & MILLER, G.A. (1980). Macrophage heterogeneity in tumour resistance: Cytostatic and cytotoxic activity of *Corynebacterium parvum*-activated and proteose peptone-elicited rat macrophages against Moloney sarcoma tumour cells. *Cell. Immunol.*, **50**, 153.

CLINE, M.J. & LEHRER, R.I. (1968). Phagocytosis by human monocytes. *Blood*, **32**, 423.

EVANS, R. (1971). Phagocytosis of murine lymphoma cells by macrophages. I. Factors affecting *in vitro* phagocytosis. *Immunology*, **20**, 67.

EVANS, R. (1979). Host cells in transplanted murine tumours and their possible relevance to tumour growth. *J. Reticuloendothel. Soc.*, **26**, 427.

EVANS, R. & BOOTH, C.G. (1976). Inhibition of $^{125}$IUdR incorporation by supernatants from macrophage and lymphocyte cultures: A cautionary note. *Cell. Immunol.*, **26**, 120.

GATELY, M.K. & MARTZ, E. (1981). Early steps in specific tumour cell lysis by sensitized mouse T lymphocytes. V. Evidence that manganese inhibits a calcium-dependent step in programming for lysis. *Cell. Immunol.*, **61**, 78.

GLENNIE, M.J. & STEVENSON, G.T. (1982). Univalent antibodies kill tumour cells *in vitro* and *in vivo*. *Nature*, **295**, 712.

GORDON, J., ROBINSON, D.S.F. & STEVENSON, G.T. (1981). Antigenic modulation of lymphocytic surface immunoglobulin yielding resistance to complement-mediated lysis. I. Characterization with syngeneic and xenogeneic complements. *Immunology*, **42**, 7.

GORDON, J. & STEVENSON, G.T. (1981). Antigenic modulation of lymphocytic surface immunoglobulin yielding resistance to complement-mediated lysis. II. Relationship to redistribution of the antigen. *Immunology*, **42**, 13.

GRIFFIN, F.M., Jr., GRIFFIN, J.A. & SILVERSTEIN, S.C. (1976). Studies on the mechanism of phagocytosis. II. The interaction of macrophages with anti-immunoglobulin IgG-coated bone marrow-derived lymphocytes. *J. Exp. Med.*, **144**, 788.

GYÖNGYOSSY, M.I.C., LIABEUF, A. & GOLSTEIN, P. (1979). Cell-mediated cytostasis: a critical analysis of methodological problems. *Cell. Immunol.*, **45**, 1.

HOGG, N. & BALKWILL, F.R. (1981). Species restriction in cytostatic activity of human and murine monocytes and macrophages. *Immunology*, **43**, 197.

KELLER, R. (1973). Cytostatic elimination of syngeneic rat tumour cells *in vitro* by nonspecifically activated macrophages. *J. Exp. Med.*, **138**, 625.

KOREN, H.S., ANDERSON, S.J. & ADAMS, D.O. (1981*b*). Studies on the antibody-dependent cell-mediated cytotoxicity (ADCC) of thioglycollate-stimulated and BCG-activated peritoneal macrophages. *Cell Immunol.*, **57**, 51.

KOREN, H.S., MELTZER, M.S. & ADAMS, D.O. (1981*a*). The ADCC capacity of macrophages from C3H/HeJ and A/J mice can be augmented by BCG. *J. Immunol.*, **126**, 1013.

KRAHENBUHL, J.L., LAMBERT, L.H., Jr. & REMINGTON, J.S. (1976). Effects of *Corynebacterium parvum* treatment and *Toxoplasma gondii* infection on macrophage-mediated cytostasis of tumour target cells. *Immunology*, **31**, 837.

MACLENNAN, I.C.M., LOEWI, G. & HOWARD, A. (1969). A human serum immunoglobulin with specificity for certain homologous target cells, which induces target cell damage by normal human lymphocytes. *Immunology*, **17**, 897.

MATSUNAGA, K., MASHIBA, H. & GOJOBORI, M. (1980). Cytostatic activity of *in vitro* activated human adherent cells against human tumour cell lines. *Gann*, **71**, 73.

NADEL, E.M. (1977). History and further observations (1954–1976) of the $L_2C$ leukaemia in the guinea pig. *Fed. Proc.*, **36**, 2249.

NATHAN, C.F., BRUKNER, L.H., KAPLAN, G., UNKELESS, J.C. & COHN, Z.A. (1980). Role of activated macrophages in antibody-dependent lysis of tumour cells. *J. Exp. Med.*, **152**, 183.

NATHAN, C.F., BRUKNER, L.H., SILVERSTEIN, S.C. & COHN, Z.A. (1979*a*). Extracellular cytolysis by activated macrophages and granulocytes. I. Pharmacologic triggering of effector cells and the release of hydrogen peroxide. *J. Exp. Med.*, **149**, 84.

NATHAN, C.F., SILVERSTEIN, S.C., BRUKNER, L.H. & COHN, Z.A. (1979*b*). Extracellular cytolysis by activated macrophages and granulocytes. II. Hydrogen peroxide as a mediator of cytotoxicity. *J. Exp. Med.*, **149**, 100.

NATHAN, C.F. & TERRY, W.D. (1977). Decreased phagocytosis by peritoneal macrophages from BCG-treated mice. Induction of the phagocytic defect in normal macrophages with BCG *in vitro*. *Cell. Immunol.*, **29**, 295.

NELSON, D.S. (1981). Macrophages: Progress and problems. *Clin. Exp. Immunol.*, **45**, 225.

NISONOFF, A., HOPPER, J.E. & SPRING, S.R. (1975). *The Antibody Molecule*. Academic Press, New York, p. 322.

PASTERNACK, G.R., JOHNSON, R.J. & SHIN, H.S. (1978). Tumour cell cytostasis by macrophages and antibody *in vitro*. I. Resolution into contact-dependent and contact-independent steps. *J. Immunol.*, **120**, 1560.

PORATH, J., AXÉN, R. & ERNBACKS, S. (1967). Chemical coupling of proteins to agarose. *Nature*, **215**, 1491.

SANDERSON, C.J. & THOMAS, J.A. (1978). A comparison of the cytotoxic activity of eosinophils and other cells by $^{51}$chromium-release and time lapse microcinematography. *Immunology*, **34**, 771.

SCHLAGER, S.I., OHANIAN, S.H. & BORSOS, T. (1979). Synthesis of specific lipids associated with the hormone-induced resistance of tumour cells to humoral immune killing. *J. Immunol.*, **122**, 108.

SCHWARTZ, B.D., KASK, A.M., PAUL, W.E. & SHEVACH, E.M. (1976). Structural characteristics of the alloantigens determined by the major histocompatibility complex of the guinea pig. *J. Exp. Med.*, **143**, 541.

STADECKER, M.J. & UNANUE, E.R. (1979). The regulation of thymidine secretion of macrophages. *J. Immunol.*, **123**, 568.

STEVENSON, F.K. & ELLIOTT, E.V. (1978). Mediation of cytotoxic functions by classes and subclasses of sheep antibody reactive with cell surface immunoglobulin idiotypic and constant region determinants. *Immunology*, **34**, 353.

STEVENSON, F.K., ELLIOTT, E.V. & STEVENSON, G.T. (1977*a*). Some effects on leukaemic B lymphocytes of antibodies to defined regions of their surface immunoglobulin. *Immunology*, **32**, 549.

STEVENSON, G.T., ELLIOTT, E.V. & STEVENSON, F.K. (1977*b*). Idiotypic determinants on the surface immunoglobulin of neoplastic lymphocytes: A therapeutic target. *Fed. Proc.*, **36**, 2268.

YAM, L.T., LI, C.Y. & CROSBY, W.H. (1971). Cytochemical identification of monocytes and granulocytes. *Am. J. Clin. Pathol.*, **55**, 283.

YAMAZAKI, M., SHINODA, H., SUZUKI, Y. & MIZUNO, D. (1976). Two-step mechanism of macrophage-mediated tumour lysis *in vitro*. *Gann*, **67**, 741.